# Evaluation of sterile glove usage on digital tactile sensitivity using the Grating Orientation Task

**DOI:** 10.3389/fvets.2024.1401130

**Published:** 2024-06-19

**Authors:** Thomas O. Riegel, Eric M. Zellner, Cheryl S. Hedlund, Karl H. Kraus

**Affiliations:** Department of Veterinary Clinical Sciences, Iowa State University, Ames, IA, United States

**Keywords:** sterile glove, surgical glove, double-gloving, tactile sensitivity, Grating Orientation Task

## Abstract

**Introduction:**

Surgical glove use may be associated with a decrease in tactile sensitivity, with thicker gloves or double-gloving techniques further altering sensation. This study evaluates digital tactile sensitivity by use of a Grating Orientation Task (GOT) with multiple sterile gloving techniques (no gloves, single standard gloving, double standard gloving, orthopedic gloves, and micro-thickness gloves).

**Methods:**

Each participant performed the GOT at increasing grating widths until correctly noting orientation in ≥8 of 10 trials with multiple glove types or double-gloving technique. Glove order was randomly assigned and participants were blinded to the orientation and dome size.

**Results:**

All gloves except micro-thickness gloves showed increased threshold sensitivity values (i.e. worse fingertip sensitivity) when compared to control (micro:control, *p* = 0.105, others:control, *p* < 0.05). Single-layer gloves showed no significant difference in sensitivity when compared to orthopedic (*p* = 0.06) or double-layer latex gloves (*p* = 0.26).

**Discussion:**

Standard latex gloves decreased fingertip sensitivity when evaluated with the GOT. Double-layer and orthopedic latex gloves do not decrease sensitivity when compared with single-layer gloving. Micro-thickness gloves may provide similar tactile sensitivity to no surgical glove.

## Introduction

Single use sterile gloves have been used in human and veterinary surgery since the 1960's, and styles of gloves have rapidly expanded and developed in the recent past. Sterile gloving techniques are used to maintain a sterile working field, minimize contamination, protect surgeons' hands from injury, and to preserve tactile sensation. Double gloving is currently recommended for most human medical procedures, as it has been shown to decrease perforation rate and potential exposure to pathogens (1). Perforation of the outer glove still occurs at a similar rate to single-gloving techniques, but inner-glove perforation is considerably lower (2, 3). While the benefits of double gloving have been shown to include a reduction in inner glove perforation, this has not directly translated to a demonstrable decrease in incision site infection rate in veterinary medicine (4). Further, some surgeons claim that double gloving techniques come with a perceived loss of tactile sensitivity and dexterity (5).

Many studies have investigated the prevalence and effect of glove puncture identified during routine surgeries in both the human and animal medical fields (6–9). There are options available for reducing the risk of puncture and exposure that range from double gloving recommendations, use of color indicator primary layer gloves, or use of woven steel protective outer gloves (2, 9, 10). Previous studies have shown no loss of dexterity or tactile sensation using the double gloving technique when evaluated with a two-point discrimination test (5). Newer testing methods to evaluate tactile sensation have been evaluated (11–13) and may provide more specific insight to a surgeon's tactile sensation with different gloving techniques.

The Grating Orientation Task (GOT) has been proposed as a method for reducing stimulus variability when assessing for tactile sensation (13). A contact dome covered in grooves and ridges of equal width can provide a larger surface and may provide better tactile sensation than a focal point analysis object. Additionally, both the orientation of the grating and the size of the grooves can be altered to assess for the limits or capabilities of tactile sensation in many applications. Previous evaluations have determined that two orthogonal orientations to the grating (proximal-distal and lateral-medial) provides adequate variability between the patterns for differentiation when assessing fingertip tactile sensation (13).

The objective of this study was to analyze the effect of different glove types and double standard gloving on the tactile sensation of participants' fingertips. The hypothesis was that there would not be a significant change in tactile sensation between the control (no glove) group when compared to the double-gloved sample group.

## Materials and methods

**Figure 1:**
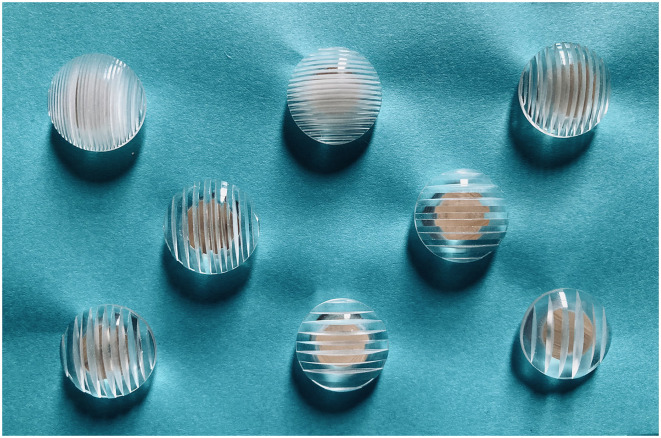
JVP domes (Stoelting Co.), sizes ranging from 0.35 mm (top left) to 3.0 mm (bottom right) in vertical and horizontal orientations.

Institutional Review Board approval was granted for the project (ISU IRB #17-563-000). Clinical-year Veterinary students, Veterinary residents, and faculty of the Department of Veterinary Clinical Sciences volunteered to perform the GOT using JVP domes (Johnston, VanBoven, and Philips domes; Stoelting, Co. Wood Dale, IL) to discern grating orientation (Figure 1). Sizes of JVP Domes used included 4.5, 4.0, 3.5, 3.0, 2.5, 2.0, 1.5, 1.0, and 0.75 mm width of grates. Gloving techniques evaluated included single latex gloving, double latex gloving, single orthopedic gloving, and micro-thickness gloves (Ansell Perry Style 42 [for both single and double-layer], Ansell Encore Latex Orthopedic, and Ansell Encore Latex Micro; Ansell LTD. Iselin, NJ). Individuals with a known latex allergy or those with previous medical conditions that could affect digital tactile sensitivity (e.g., carpal tunnel syndrome, diabetes mellitus with, or without neuropathy, etc.) were excluded from participation. All double gloving techniques were evaluated with both the inner and outer glove being of the same selected size for each individual participant, with size being determined by the participant. Glove sizes were used based on each individual participant's self-selection, and no direct size guidance was given. Glove sizes used included 6.0 through 8.0 for all standard latex or micro-thickness gloves. Orthopedic gloves varied in size from 6.5 through 8.0. All individuals who used size 6.0 gloves for standard and micro-thickness testing used size 6.5 for orthopedic gloves due to size limitations from the manufacturer for the specific style selected for this study. Control data was collected from each individual participant without the use of surgical gloves before performing the GOT for any gloved categories. The gloved tests were performed in a modified randomization pattern, where no glove was used first, and then the remaining gloves were tested randomly. However, single standard gloving was then always followed by double standard latex wherever the former fell in the previously randomized order.

The Grating Orientation Task (GOT) was used to evaluate digital sensitivity via participants' detection of correct orientation of the device. The same researcher performed all GOT trials (TR) to provide consistency in placement and pressure of dome to participants' fingertip. Participants' vision was blinded via a cardboard screen to obscure dome size and orientation. Each participant placed their hand through the screen and held their dominant hand supine with the index finger extended. Grates were placed on a participants' dominant index finger oriented in either a proximal-distal (vertical) or lateral-medial (horizontal) orientation for 10 touches at each grating size selected. Orientation for each individual test was determined via random generation of a coin flip (Random.org coin flipper tool) with heads being assigned the vertical orientation and tails the horizontal orientation of grating. The correct verbal identification of orientation (horizontal vs. vertical) of the grating required 80% or better success by the participant to determine a threshold sensitivity value, based on manufacturer recommendation of >50% above random chance. If three incorrect responses were recorded for any size grating, that was determined to be too small of a size grate for threshold sensitivity for that participant, and the next larger dome size was then evaluated. Each participant completed a total of 10 touches for a given size grating, even after they had failed to identify proper orientation in 3 or more touches, in an effort to avoid knowledge of incorrect answers. Participants first performed the task with the 1.5 mm size dome, and then would move to either a smaller size if their responses were correct in at least 8/10 touches, or would move to an incrementally larger size grating if incorrect identification of orientation was determined. A response of “unknown” or “unsure” was recorded as an incorrect response. If a participant attempted to roll or press on the dome at a pressure beyond what the evaluator supplied, this individual touch was discarded and a sequential touch was used to fulfill the total required for each size after generating another random coin flip for orientation. This was continued until the participant correctly identified the orientation of the grating in at least 8/10 touches, which was then recorded as their threshold sensitivity for each glove test parameter. Participants were not notified of the proper orientation after each touch and were not told their overall threshold grating size for each gloving technique.

### Analysis

A power analysis was calculated to determine the sample size needed to detect a 0.5 mm threshold difference in the population above the mean. Alpha set at 0.05 with a power of 0.8 showed a minimum size of 53 participants was required for significance. Data was statistically analyzed using online statistical analysis software (*Prism*, GraphPad Software, http://www.graphpad.com). Normal distribution was not found via use of a Shapiro-Wilk test. For each independent recorded variable, a mean, standard deviation, and standard error from the mean were calculated. Glove threshold values were compared with a Kruskal-Wallis test for multiple independent samples. Significance was assessed between groups with the Dunn multiple comparisons test, and significance was set at *p* < 0.05. A *post-hoc* analysis was performed using a Mann-Whitney u-test.

## Results

**Figure 2:**
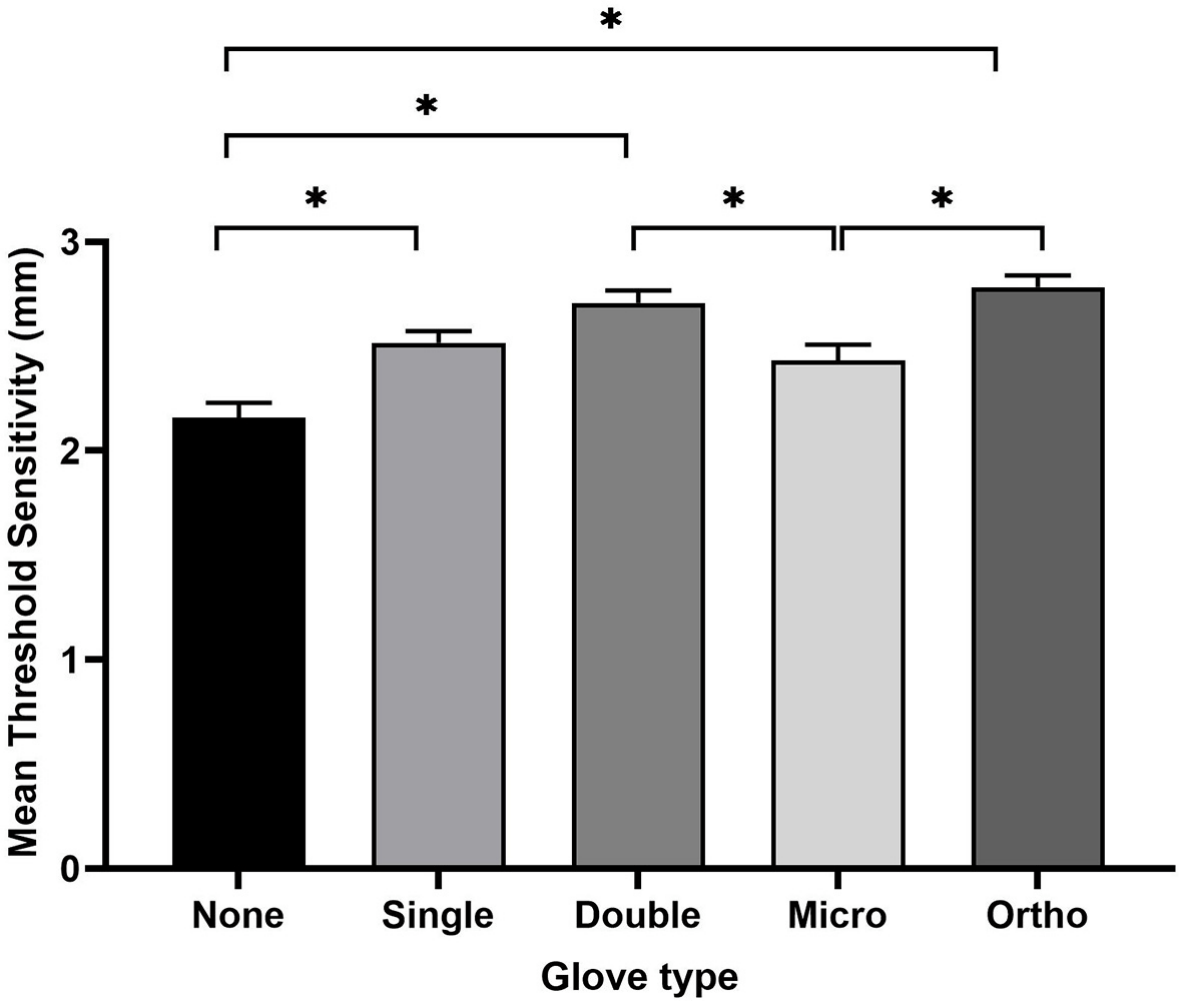
Threshold sensitivity values (mean ± standard error from the mean) for each gloving category evaluated. Asterisks indicate a statistically significant difference in threshold sensitivity between groups (*p* < 0.05).

A total of 60 participants were enrolled in and completed the study. Fifty-seven participants were 4^th^ year clinical veterinary students, two were small animal surgery residents, and one was small animal surgery faculty. Mean age of participants was 25.8 years (range 24–34); gender was not recorded. A total of 53 participants were right-hand dominant and 7 participants were left-hand dominant. Median surgical glove size was 7. The mean GOT threshold value without gloves was 2.16 ± 0.53 mm, single-layer latex 2.52 ± 0.43 mm, double-layer latex 2.71 ± 0.45 mm, micro latex 2.43 ± 0.58 mm, and orthopedic latex 2.78 ± 0.43 mm (Figure 2).

The non-gloved test compared to micro-thickness gloves showed no statistically significant difference in threshold sensitivity, *p* = 0.105. Single-layer latex showed an increased fingertip threshold sensitivity when compared to no gloves, *p* = 0.01. Both double-layer latex and orthopedic gloves showed an increased threshold value when compared to no gloves, *p* < 0.001 for both glove types. There was no statistically significant effect when comparing the threshold sensitivity of single-layer gloves to either double-layer or orthopedic gloves, *p* = 0.27 and *p* = 0.06, respectively. Double-layer and orthopedic gloves did have a statistically significant higher GOT threshold value (decreased fingertip sensitivity) when compared to micro-thickness gloves, *p* = 0.04 and *p* = 0.005, respectively. There was no difference between single-layer or micro-thickness gloves, *p* = 0.93. There was no difference in threshold sensitivity between double-layer or orthopedic gloves, *p* = 0.97. No statistically significant differences were noted when evaluating left-handed participants' threshold values vs. right handed participants' values for any glove types tested.

## Discussion

The objective of this study was to evaluate the sensitivity threshold for different gloving techniques by use of the GOT. The perception that double-layer latex gloves may decrease fingertip sensitivity when compared with single-layer latex gloves was not supported, as they had similar threshold sensitivity values. However, use of single-layer standard latex gloves did show an increased threshold value when compared to no gloves, suggesting a poorer fingertip sensitivity. The use of single-layer latex surgical gloves increased GOT threshold values by 16.7% when compared to the control. The use of double-layer and orthopedic latex gloves increased threshold values by 25.4% and 28.7%, respectively. However, no significant difference was noted between the single-layer, double-layer, and orthopedic glove groups. Micro-thickness latex gloves had a 11.7% increased threshold sensitivity relative to the control, but this was not statistically significant. As an option advertised for improved tactile sensitivity with a 20% thinner latex glove (14), micro-thickness gloves appear to yield similar sensitivity to no glove use (*p* = 0.105). To the authors' knowledge, these thinner style gloves have not been shown to have an increased perforation rate, but studies comparing specific glove styles and perforation rates are lacking. Additionally, while limited studies have evaluated different brands of surgical gloves (15), our study evaluated multiple thickness styles as well as double vs. single gloving within one glove manufacturer. Orthopedic gloves have been shown to have a perforation rate similar to double-gloving techniques in veterinary medicine (16) and are advertised as up to 50% thicker than standard latex surgical gloves (17). However, one study has shown a decrease sensitivity with use of orthopedic gloves while showing a similar perforation rate in human arthroplasty between orthopedic-thickness and single-layer standard latex gloves (18). Our study showed a similar threshold sensitivity value for both standard latex and orthopedic gloves when evaluated using the GOT.

While the GOT has been used in multiple settings to evaluate digital tactile sensitivity, to the authors' knowledge it has not been used to evaluate sensitivity with surgical gloves. The two-point discrimination task is more commonly used in similar studies (5, 9, 18), the GOT provides a large platform for assessing fingertip sensitivity and could provide a more accurate interpretation in a surgical setting. Our study found similar results to the conclusions of Fry et al. when evaluating tactile sensitivity of single vs. double gloving (5).

Sterile surgical gloves are a necessity in modern veterinary surgery. With the risk of glove perforation or damage well-documented, recommendations can be made for either thicker gloves or double-layer gloves in those procedures with higher risk of perforation (2, 3, 6, 9, 10). Based on evaluation with the GOT, the use of sterile latex surgical gloves does increase GOT threshold sensitivity value. However, this effect on threshold sensitivity was not found to be significant when comparing standard gloving to orthopedic gloves or double-layer gloves. Recommendations for these gloving techniques that could decrease risks with glove perforation could reasonably be made without significant concern for loss of tactile sensitivity when compared to standard gloving.

The authors acknowledge several limitations to the study. A spring or pressure sensor was not used, which could change the sensitivity threshold for some participants when evaluating with the GOT (12). Fit of the gloves being used was not measured or evaluated and has been shown to affect dexterity, although not sensitivity, with improper sizing (19). Glove sizes used were based on individual's selection rather than fitting to a specific size. The duration of glove wearing prior to testing, as well as the fit of sterile surgical gloves could be another route of future investigation to determine those effects on fingertip sensitivity when evaluated with the GOT. Additionally, gender was not recorded in an effort to blind evaluation of the data interpretation, but could be a source of variance in the accuracy of results or fingertip sensitivity. Further, the entire population selected for study is one that has experience and familiarity with sterile gloves. A sample evaluating participants who have infrequent or no exposure to sterile gloving techniques may provide a more accurate assessment of the impact on fingertip threshold sensitivity values.

Participants were instructed to not roll or move their finger, and this was not deemed feasible without direct researcher oversight controlling and censoring those tests where participants did inadvertently roll or move their fingertip. Those individual test results where improper technique required correction were not recorded, and the test was repeated until each individual had an appropriate number of touches to determine threshold sensitivity (≥8 positive responses out of 10 touches at a set dome size). Participants were instructed to invert their hand and hold in a supinated neutral position. However, this was not specifically controlled in regards to wrist angle or hand posture, with some incorrect postures potentially affecting tactile sensitivity (20). Since data collection for this study, a more standardized guideline of instruction and positioning of the participant's hand during the GOT has been proposed. Wang et al. proposed a stepwise “two-down one-up” rule to more specifically determine an exact threshold sensitivity value (21). This consisted of a decrease in grating size tested after two correct responses, or increasing width with one incorrect answer. A step up was recorded as a transition point, and the test evaluated the mean of 8 of these transitions to determine threshold sensitivity value. Further, Wang et al. (21) established a short teaching parameter to visualize and confirm grating orientation of the JVP domes before being tested, which was not performed in the present study. In our study participants threshold sensitivity value was only assessed on a total of 10 touches per dome size, but this allowed for more tests to be completed in a given timeframe, as the test was performed to determine a value for each of the five tested parameters.

A limited selection of glove types were used in this study, and those selected for testing were due to the authors' experiences with common glove types used in a veterinary teaching hospital setting. This did also limit the specific sizes of gloves available, with some participants needing to perform the test with orthopedic gloves $\frac{1}{2}$ size larger than with other sizes of gloves, due to manufacturer limitations of sizes and styles available. Further studies could expand upon other commonly used glove types (e.g., nitrile, latex-free surgical gloves, textured) to determine their effect on fingertip sensitivity under similar testing parameters.

The GOT test used in this study is simply a touch and tactile sensitivity test. It does not evaluate dexterity and does not evaluate motion, friction, or changes in pressure that may have a larger impact on more minute fingertip sensitivity (13). The change in fingertip sensitivity noted in this study may not correlate to an altered sensitivity in an *in vivo* setting, where much more information is available to discern small changes in surface texture.

In conclusion, single-layer, double-layer, and orthopedic sterile latex gloves showed an increased threshold sensitivity value when compared to the bare fingertip. However, this difference was fairly small, and there was no difference in sensitivity between single-layer and double-layer latex gloves. While there may not be the same impetus for the strong recommendation of double gloving in the veterinary field when compared to human medicine, it may still have use in procedures with a high rate of glove perforation. This study suggests that double gloving may be recommended as deemed necessary without significant concern for its impact on fingertip sensitivity.

## Data availability statement

The original contributions presented in the study are included in the article/supplementary material, further inquiries can be directed to the corresponding author.

## Ethics statement

The studies involving humans were approved by the Iowa State University: Institutional Review Board. The studies were conducted in accordance with the local legislation and institutional requirements. The participants provided their written informed consent to participate in this study.

## Author contributions

TR: Conceptualization, Data curation, Funding acquisition, Writing – original draft, Writing – review & editing. EZ: Writing – review & editing. CH: Conceptualization, Writing – review & editing. KK: Writing – review & editing.
